# The place for prophylactic cerclage in the infertile patient with established cervical incompetence who conceived twins after septum reduction.

**Published:** 2017-06

**Authors:** Jennifer Deanna, Omar Abuzied, Fahmi Islam, Ivana Vettriano, Frederico Rocha, Mostafa Abuzeid

**Affiliations:** Department of Obstetrics and Gyneacology, Genesys Regional Medical Center, Michigan State University Statewide Campus System, Grand Blanc, MI; Department of Obstetrics and Gyneacology, Hurley Medical Center, MSU College of Human Medicine (Flint Campus), Flint, MI; Division of Maternal Fetal Medicine, St. John Providence Hospital/ Michigan State University, South eld, MI, USA; Division of Maternal Fetal Medicine, Department of Obstetrics and Gyneacology, Hurley Medical Center, MSU College of Human Medicine (Flint Campus), Flint, MI; Division of Repro- ductive Endocrinology and Infertility, Department of Obstetrics and Gyneacology, Hurley Medical Center, MSU College of Human Medicine (Flint Campus), Flint, MI

**Keywords:** Twin gestation, cerclage, uterine septum, cervical incompetence, IVF, prophylactic

## Abstract

**Introduction:**

It is well known that cervical incompetence and associated preterm birth confers greater morbidity and mortality on birth outcomes, with an additional increased risk of cervical incompetence in higher order gestations. While the pathophysiology of cervical incompetence has yet to be elucidated, research has identified risk factors and assessed outcomes of numerous interventions. Cervical cerclage has been shown, in certain situations involving singleton pregnancies, to improve outcomes. Conversely, rescue cerclage increases the risk of preterm birth in twin gestations. However, these studies did not consider the unique situation of infertile patients, with known cervical incompetence, who have utilized assisted reproductive technologies to attain pregnancy. This study aims to describe the outcomes of infertile patients with known cervical incompetence, carrying twin gestation, who have undergone cervical cerclage.

**Methods:**

This case series includes eight infertile patients who have cervical incompetence resulting in fetal loss between 20-24 weeks after in vitro fertilization embryo transfer (IVF-ET). These patients continued with IVF treatments and subsequently conceived twins. All patients underwent prophylactic cervical cerclage placement before 12 weeks. The outcomes of these pregnancies are reviewed.

**Results:**

All pregnancies resulted in the delivery of viable twins. Six of the eight pregnancies (75%) were carried beyond 34 weeks. One pregnancy delivered at 31 weeks and one pregnancy delivered at 25 weeks after placental abruption.

**Conclusions:**

This data suggest that the use of prophylactic cervical cerclage may be beneficial in improving reproductive outcomes in infertile patients with known cervical incompetence that subsequently conceived twin gestations via IVF-ET treatment.

## Introduction

Preterm birth confers great morbidity and mortality in obstetrics, with preterm birth being the leading cause of neonatal mortality, the cause of 70% of neonatal deaths, 36% of infant deaths, and 25-50% of long-term neurologic impairment (Berghella et al., 2011). In an effort to mitigate the associated morbidity and mortality, a large breadth of research has been devoted to identifying risk factors of preterm birth. Some of these identified factors for preterm birth and losses include inflammation, infection, uterine over distension, and cervical insufficiency (Institute of Medicine, 2007).

Cervical insufficiency, which is characterized by painless cervical dilation resulting in second trimester miscarriage, presents the patient-physician dyad with a great hurdle to overcome in order to achieve a successful pregnancy. This difficulty is compacted by the fact that the pathophysiology of cervical incompetence has not come to full fruition. Although the pathophysiology of cervical insufficiency has yet to be completely elucidated, several risk factors have been identified. These risk factors include cervical trauma (Vyas et al., 2006), cervical dilation for procedures (Shah and Zao, 2009), uterine anomalies (Chan et al., 2011), and collagen disorders (Iwahashi et al., 2003).

Once cervical insufficiency is identified, several interventions have been trialed to decrease the risk of preterm delivery. Some of these interventions include cerclage, pessary, progesterone, lifestyle intervention, antibiotics. Of these interventions, cerclage is found to improve outcomes, albeit in very specific situations. The situation in which cerclage is recommended is in women with singleton pregnancies with a history of a preterm delivery and a shortened cervix before 24 weeks (ACOG Bulletin, 2014).

In 2011, a meta-analysis of randomized trials of women with prior spontaneous preterm birth carrying a singleton gestation had placement of cerclage after being found to have a shortened cervix before 24 weeks. Results yielded a total reduction in neonatal morbidity and mortality by one third (Berghella et al., 2011). The current cerclage indications include “history indicated cerclage” which applies to women with a history of two or more consecutive second trimester losses or three or more preterm births (less than 34 weeks) and “ultrasound indicated cerclage”, which is recommended in a patient with one prior second trimester loss or one or two preterm births plus cervical length less than 25mm before 24 weeks. Additionally, cerclage is beneficial based on physical exam in a patient with a singleton gestation presents with a dilated cervix (Rust and Odibo, 2014).

However, the above data and recommendations are only in regard to women with singleton gestations. In fact, ACOG does not recommend cerclage for twin gestations, as data on cerclage in twins is scant and some data show increased morbidity with cerclage placement in twins (Hayes, 2014). A 2014 review of data did not find improved outcome in the placement of rescue cerclage in twin gestations and some show an increase in morbidity (Rafael et al., 2014). However, the number of subjects in these studies is small. Furthermore, women with known cervical incompetence seeking assisted reproductive technologies (ART) are not specifically studied. This is a subpopulation of women who are often faced with multiple of the aforementioned risk factors for preterm delivery at baseline. This risk is then amplified when the patient conceives twins via ART. Navigating these risk factors while providing artificial reproductive technologies, in hopes to increase the likelihood for a successful pregnancy, is paramount to assist these women to achieve the successful pregnancy they so desire.

This importance is highlighted by the fact that approximately 1.5 % of all births are a result of assisted reproductive technologies (Ory, 2013). Additionally, IVF resulting in multiple gestations account for 20% of all multiple births. Although, the percentage of multiple births from ART has been declining since 2009, recent data from the CDC indicates that 44 % of ART infants were part of a multiple gestation (Kulkarni et al., 2013).

Another risk factor of cervical incompetence often found in women with infertility is the presence of a uterine anomaly. Given that a uterine septum is the most common uterine anomaly, many women who seek ART have a uterine septum. This poses another risk factor for cervical incompetence and could put a pregnancy at great risk.

With the multitude of risk factors for cervical incompetence which are often seen in patients with infertility, it is of utmost importance to work toward filling this informational void in order to provide the patient with as much information to increase their likelihood for a successful pregnancy. Again, this is especially true when considering the patient with a history of cervical incompetence who is carrying twins.

This study aims to describe the outcomes of the subset of patients with infertility, known cervical incompetence, following hysteroscopic correction of uterine septum, who are carrying twins and have undergone cervical cerclage.

## Methods

**Figure 1:**
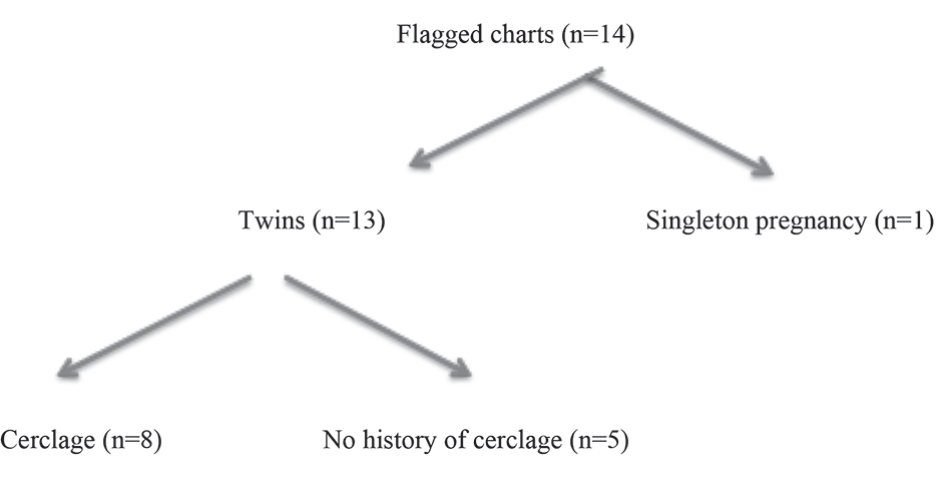
— Flow sheet for chart review

We retrospectively analyzed charts from 2006-2014 of patients with previously established cervical incompetence after hysteroscopic septum correction, who subsequently conceived twins via IVF and chose to undergo prophylactic cervical cerclage. Charts were flagged for patients with history of septum reduction who conceived twins. These charts were reviewed. One was excluded as the subsequent pregnancy was actually singleton. Additionally, charts were excluded if the patient did not undergo cerclage (Figure 1). Gestational ages at delivery were compared for each patient for their pregnancy without cerclage and pregnancy with cerclage. Additionally, pregnancy outcomes associated with the cerclage pregnancy were analyzed. These outcomes included reported pregnancy complications and infant birth weights. These outcomes were self-reported by the patient after delivery. The diagnosis of cervical incompetence was diagnosed by the patient’s obstetrician at time of loss.

Our sample size is small. This is a function of our inclusion criteria. Patients were included because they had a history of septum reduction, followed by poor obstetric outcomes of miscarriages attributed to cervical incompetence. Subsequently, the patients conceived twins through IVF for reasons related to unsuccessfully single embryo transfers and poor obstetric outcomes. The decision to transfer multiple embryos was done after extensive counselling with the patient, reviewing risks of multiple gestations, likelihood of success, and weighing this with the patient’s feelings of loss and increasing feelings of hopelessness and great desire for a successful pregnancy.

## Results

All pregnancies resulted in the birth of viable twins. Six of the eight pregnancies (75%) were carried beyond 34 weeks (Table I). One pregnancy delivered at 31 weeks and one pregnancy delivered at 25 weeks after placental abruption. This placental abruption was the only self reported pregnancy complication. The mean gestational age at time of delivery without cerclage was 21 weeks, where as with a cerclage in situ, the average gestational age at time of delivery was 34 weeks (Table I). The range of birth weights after cerclage was 710 grams to 3460 grams.

**Table I:** Pregnancy information of charts revieuwd. FET=frozen embryo transfer; ICSI=intracytoplasmic sperm injection

Pt #	GA of delivery without Cerclage	GA of delivery with Cerclage	Route of Cerclage Placement	Septum Division	ART method	Number of embryos transferred	Birth weights	Pregnancy Complications
1	25 weeks	31 1/7 weeks	Vaginal	Yes	FET	3	A: 1450g; B: 1330g	None
2	20 weeks	25 weeks	Vaginal	Yes	ICSI	2	A: 910g B: 710g	Placental abruption
3	20 weeks	37 3/7 weeks	Trans abdominal	Yes	ICSI	2	A: 2750g; B: 2920g	None
4	19 weeks	36 weeks	Vaginal	Yes	ICSI	2	A: 2580g; B: 1960g	None
5	18 weeks	38 weeks	Vaginal	Yes	ICSI	2	A: 3260g; B: 3460gr	None
6	22 weeks	35 weeks	Trans abdominal	Yes	FET	3	A: 2130g; B: 1980g	None
7	27 weeks	35 weeks	Vaginal	Yes	FET	2	A: 3010g; B: 3400g	None
8	22 weeks	35 3/7 weeks	Vaginal	Yes	FET	2	A: 2830g; B: 2640g	None

## Discussion

The data from this study strongly suggest that the use of prophylactic cervical cerclage may be beneficial in improving reproductive outcomes in infertile patients with known cervical incompetence and status post hysteroscopic surgical correction of uterine septum that subsequently conceived twin gestations via IVF-ET treatment. Each patient suffered pregnancy loss due to cervical insufficiency. Subsequently, each patient conceived twins by IVF and chose prophylactic cerclage placement. Interestingly, cerclage placement resulted in 100% of patients delivering viable twin pregnancies. Of those 75% of patients carried the pregnancy beyond 34 weeks. It is known that rescue cerclage placement results in worse outcomes in twins. Our cerclage placement was prophylactic. This raises the question about the difference in efficacy of prophylactic versus rescue cerclage in twins.

Perhaps the increased stress on the rescue cerclage or that the cervix shortens more quickly accounts for the worse outcomes after rescue cerclage placement. This further supports the notion that more research is needed on the use of cerclage and timing of cerclage placement in twins.

Furthermore, data suggests 5-10% of the patients with uterine anomalies have an element of cervical incompetence. Unfortunately it cannot predict preterm birth due to cervical incompetence and if it will happen again. Therefore this data advocate for the use of history based prophylactic cerclage to give the pregnancy the highest chance of success, especially in this specific patient population who have been through heartbreak with poor obstetric outcomes and repeated unsuccessful IVF cycles. Again, the majority of current research has not shown a benefit to placement of cerclage in twin gestations in general. However, these data do not evaluate this specific subset of patients with infertility that is carrying twin gestations in the face of known cervical incompetence and status post hysteroscopic septum reduction. Furthermore, a study in 2013 by Mamas that evaluated the effect of prophylactic cerclage in women who conceived twins and triplets after fertility treatment did reveal a benefit (Mamas et al., 2013). This study did not specifically evaluate women who had a known uterine septum, status post resection, which is the subset population assessed with our data set. Our population subset represents a gap in research that begs further research, especially, when it is considered that use of reproductive technologies continues to increase and a large percentage of multiple gestations are attributed to ART. Great attempts to mitigate the increase in multiple gestations via ART have been made. In 2013, ASRM released a committee opinion giving recommendations to limit the number of embryos transferred. Additionally, data are increasing regarding the success of single embryo transfer. However, the situation of multiple gestations in the setting of ART remains a reality. Therefore, there is a great need to increase the breadth of research with regard to cerclage placement in this patient population. A more robust research base in this area will allow providers to advise the patient with the best recommendations in hopes of a successful pregnancy. Of note, this study only included patients whose cycles ended with twin pregnancies. Given these patients had a history of cervical incompetence may beg the question as to why more than one embryo would be transferred. These patients had undergone several failed single embryo transfers. For example, Patient 3 had one fresh transfer, which was unsuccessful. She then underwent two frozen cycles with single embryo transfers, which were also unsuccessful. With 3 embryos remaining, all of which were poor quality, the decision was made to transfer all of the embryos.

Physicians aim to provide the patient with the best care and recommendations while being guided by the current literature, practice guidelines, and the pillars of medical ethics. However, given that each patient is unique, we are sometimes faced with patients that do not fit into the patient populations that research has assessed. This leads to the need to extrapolate recommendations from one situation to provide recommendations in another. Cerclage in twin pregnancies is one of those such situations. Current literature is limited in regard to cerclage in twins. In an effort to provide the best care for patients, recommendations are made from the available, albeit limited literature. This literature shows mixed results at best for cerclage placement in twins. With that, current cerclage placement indications are very specifically outlined for singleton pregnancies only. However, our data strongly suggests that some subsets of twin pregnancies might greatly benefit from cerclage and reiterates that this topic deserves more research. This is especially true in those women with twin gestations in the face of pre-existing preterm risk factors, cervical insufficiency, and who are undergoing ART. Working toward a more robust research base in this subset of patients is paramount to give these patients the best chance to maintain the pregnancy she so hopefully seeks, while staying true to the ethical standards of beneficence and non- maleficence. The data from our study suggests that the use of prophylactic cervical cerclage may be beneficial in improving reproductive outcomes in infertile patients with known cervical incompetence that subsequently conceived twin gestations via IVF-ET treatment. The main limitations of this study are the small number of subjects and that the study is a retrospective chart review of eight patient cases. Results were obtained by patient recall and follow up after delivery. However, the results strongly suggest that this is a patient population that needs to be further evaluated for possible utility of cervical cerclage to decrease preterm birth. Additionally, this data includes vaginal and trans-abdominal cerclage methods. Further research is needed to determine which mode of cerclage placement is most beneficial, as well as best timing. Furthermore, specific indications for possible cerclage placement in twin gestations would also be of great interest.
